# Unleashing implementation research to accelerate national noncommunicable disease responses

**DOI:** 10.1186/s12992-021-00790-5

**Published:** 2022-01-24

**Authors:** Tea Collins, Svetlana Akselrod, Daria Berlina, Luke N Allen

**Affiliations:** 1 World Health Organization, Room 4059, Avenue Appia 20, CH-1211 Geneva 27, Switzerland; 2 Department of Clinical Research London School of Hygiene and Tropical Medicine, London, UK

## Abstract

Noncommunicable diseases (NCDs) are the leading cause of death and disability worldwide. They exact a disproportionate toll in low and middle-income countries, and the world is not on-track to meet international targets for reductions in premature NCD mortality. Largely, we know *which policies work* for tackling NCDs, and the World Health Organization (WHO) has developed a package of ‘best buy’ policies that are highly cost effective. However, we don’t necessarily know *how* to adapt and implement these policies in new populations and cultures. Implementation Research (IR) is emerging as a potent tool for gearing the international response, providing a scientific approach to study the processes used to implement policies and interventions and the contextual factors that affect these processes. Amidst growing interest from policymakers, we identify four main areas for action: high-level engagement with IR among international NCD leaders; domestic investment in technical capacity-building; the creation of new financing streams for IR research; and the development of multi-stakeholder engagement mechanisms that can convene and leverage the perspectives and resources of multiple actors with overlapping aims.

**Supplementary Information:**

The online version contains supplementary material available at 10.1186/s12992-021-00790-5.

## Introduction

We are moving too slowly to address noncommunicable diseases (NCDs). These conditions are responsible for 74% of global mortality, and over four fifths of all premature NCD deaths occur in low and middle-income countries (LMICs) [1, 2]. The human, social and economic costs of NCDs are substantial and failure to tackle them will exert an estimated US$47 trillion drag on LMIC economies alone between 2011 and 2025 [3].

Approximately 10 million premature NCD deaths could be avoided by 2025 if governments implemented suite of cost-effective NCD policies dubbed the WHO ‘best buys’ [4] that were introduced in the 2013-2020 Global Action Plan [5]. An updated package of NCD best buy policies (Appendix 3 of the Global Action Plan) was unanimously endorsed by the World Health Assembly in 2017 [4]. These policies include tobacco taxation, bans on tobacco advertising, and mass media campaigns to promote physical activity. The seventy-second World Health Assembly extended the Global Action Plan deadline by a decade [6] to align with the 2030 Agenda for Sustainable Development [7]. Subsequent NCD progress reports [8, 9] and the Global Action Plan mid-point evaluation [10] indicate that fewer than half of the recommended NCD policies have been implemented worldwide [11]. Furthermore, the world is not on course to meet SDG target 3.4: to reduce premature NCD mortality by a third by 2030 [10].

Whilst the evidence supporting the effectiveness of population-level NCD policies continues to grow, an increasingly critical ‘know-do’ gap is emerging. We know which policies need to be implemented, but we don’t always know *how* to implement them to meet unique needs of different cultures, populations, and sociodemographic contexts. The lack of evidence on how to implement effective NCD policies is especially acute in LMICs. More than 80% of the research underpinning the best buy interventions has been performed in high-income countries, so it is not clear how effective these policies are in the populations where over 80% of NCD deaths occur [12]. New research has also shown that countries experiencing the fastest growth in NCDs are also the least likely to have implemented NCD policies [13].

A recent WHO-led Lancet editorial stressed the “urgent necessity” of harnessing ‘implementation research’ to help countries course-correct [14]. This call resonates with the Global Action Plan mid-point evaluation which found that especially poor progress had been made in the domain of ‘promoting and supporting capacity for NCD research’ in WHO Member States [10]. It is important to note that in July 2021 WHO established the Technical Advisory Group of Experts on NCD Research and Innovation to act as an advisory body to WHO to further WHO’s leadership and coordination role in promoting and monitoring global action on research and innovation to prevent and control NCDs.

There may be agreement that implementation research (IR) is needed to accelerate progress in tackling NCDs, but uncertainty remains around what is actually meant by the term, how IR can be used, and what steps need to be taken in order to promote spread and adoption. This short article answers each of these questions in turn using real-life examples.

### What is implementation research and how can it help us accelerate progress?

Simply put, implementation research is the main means of tailoring known solutions to new contexts. Early definitions in the health literature emphasised the fact that the field unites multiple disciplines around the motivating intent of understanding ‘what, why, and how’ interventions work and testing approaches to improve them.”[15] IR shares important overlaps with ‘practice-oriented research’ from the field of health economics in that the driving focus is application to practice and praxis within real-world health systems [16–18]. IR is a form of translational research, occupying the T3 and T4 domains outlined by the seminal 2013 Institute of Medicine report [19]. In the same year, the WHO Alliance for Health Policy and Systems Research released ‘Implementation Research in Health: a practical guide, defining IR as “the scientific study of the processes used to implement policies and interventions and the contextual factors that affect these processes” [20]. The discipline uses quantitative, qualitative and mixed-methods approaches to explore the factors that affect how policies may be used in real-life settings, and to identify the most efficient and cost-effective strategies for implementation. As such, IR can help us to explore the interplay between a policy and its local context; identify implementation challenges; and suggest feasible and scalable solutions for overcoming them [20].

#### Example: Using IR to optimise Malaysia’s healthy diet strategy

The International Network for Food and Obesity/NCD Research, Monitoring and Action Support (INFORMAS) is a non-governmental organisation that is committed to support the implementation of effective dietary policies. In the mid-2010s the organisation adopted an IR approach to bolster Malaysia’s national diet strategy. They started by developing a policy evaluation tool that was used to perform a baseline assessment of the government’s current food policy approach, with reference to international best practice. The resulting ‘Food Environment Policy Index’ assessment was conducted in 2017-2018 and identified a range of policy gaps. Next, the research team convened a panel of policy experts with deep knowledge and experience of the Malaysian context to develop a series of tailored measures to bolster NCD prevention (summarised in the Appendix). By studying existing policy gaps and recommending tailored policies, the research team made an important contribution to the national healthy diet strategy. In the future, further IR methods could be used to study the processes used to implement these recommendations.

IR approaches can be used to answer questions at any point along the spectrum from problem definition to programme evaluation. Box 1 provides examples of the kinds of research questions that might be asked, the types of methods that could be used, and example studies from literature.

Implementation research questions, methods, and examples:

- Which NCDs and risk factors pose the greatest threat to our population?
  - Methods: situational analyses, observational studies.
  - Example: Researchers recruited over 500,000 participants to the Chinese biobank study to understand the major causes, risk factors, and prevalence patterns of NCDs over the course of 15-20 years [21].

- Where are the policy gaps?
  - Methods: policy review, stakeholder interviews.
  - Example: An ASEAN expert review found that Southeast Asian countries were not engaging with sufficient levels in NCD surveillance activities [22].

- What is the most appropriate policy?
  - Methods: literature review, modelling, interviews, trials.
  - Example: Researchers used Turkish demographic and epidemiological data to run a macro-simulation study to identify the NCD policies that would prevent the most NCD deaths in 2017 [23].

- What barriers have prevented implementation of known solutions?
  - Methods: interviews, stakeholder analysis, political analysis.
  - Example: Researchers found that front-of-package nutrition labelling was not being implemented in Portugal because the Ministry of Agriculture was the responsible agent and did not perceive benefits for their stakeholders. Responsibility was shifted to an inter-departmental group, which promptly adopted the policy [24].
  - Example: Researchers found that a paucity of policy development personnel and an overreliance on foreign consultants was impeding the timely implementation of NCD policies in the Caribbean [25].

- Why have previous measures succeeded or failed?
  - Methods: qualitative reviews, interviews, system dynamic modelling.
  - Example: Study of Kenyan essential medicines found that cardiovascular medications were available, but underdeveloped delivery systems impeded access [26].
  - Example: Researchers used document review and key stakeholder interviews to assess the NCD policy implementation processes in five African countries, finding – among other things – that industry interference was a major barrier [27].

- How have our barriers been overcome in similar contexts?
  - Methods: interviews, literature review, case studies.
  - Example: Key stakeholders from four eastern Mediterranean countries were interviewed to identify barriers, best practices and lessons learned in developing multisectoral NCD action plans. [28]

- Whilst implementing a new policy: “For whom does it work? In what settings? And in what dose, frequency, intensity, and duration?” [29].
  - Methods: time series analyses, surveys, trials with subgroup analyses, interviews, focus groups, ethnography.
  - Example: Researchers used purchasing and demographic data to explore the impact of Mexico’s SSB tax by age, sex, and socioeconomic status, finding that the largest falls in consumption were observed among low-income households [30].

Other important implementation research questions include 'Who should be involved in or actively excluded from the policy development and implementation processes?' and ‘what is the best way to pay for multi-stakeholder implementation initiatives?’ As the examples above illustrate, virtually any research method can be used to answer IR questions. The focus should be on selecting the right method for the right question in order to discover how an established policy can be adapted to improve NCD outcomes in a particular population. The process of selecting the appropriate tools and methods can be overwhelming for those new to the field – including the majority of NCD programme managers who sit within government. Fortunately, WHO has already produced guidance on the use of IR methods in the prevention and control of NCDs [31], and the Prince Mahidol Award Conference (PMAC) commissioned book ‘Best Buys, Wasted Buys and Controversies in NCD Prevention’ also provides detailed guidance for programme managers [32]. The authors present the SEED tool (Systematic thinking for Evidence-based and Efficient Decision-making) which leads implementers through the cycle of assessing the evidence base, considering generalisability to the population in question, and assessing the economic and political environment. These processes require interdisciplinary skills, as well as collaboration between researchers, policymakers, and representatives from affected communities.

### Promoting spread and adoption through capacity-building and multistakeholder collaboration

Based on what IR can achieve, policy makers keen to understand the requirements for developing and scaling international IR capacity. This topic was covered in depth at the 2019 Prince Mahidol Award Conference in Bangkok during a full-day session hosted by the WHO Global NCD Platform, Global Alliance for Chronic Diseases, and the International Development Research Centre. Seventy stakeholders were convened from governments, academia, civil society and the private sector to advance thinking on how implementation science can be used to address NCD implementation challenges. Case studies discussed at that meeting are presented in this section.

The first step is acknowledging the need for IR amongst those charged with leading the NCD response at the national and international levels – as has been manifest in recent WHO publications [10, 14, 33] – married with a growing understanding of how IR operates. Education and training for policymakers, web events, and the creation of forums and peer-to-peer learning networks can all support these aims. The Joint Learning Network [34], GHDonline [35], the G7 Primary Health Care universal knowledge initiative [36], and the Global Coordination Mechanism *NCD Knowledge Action Portal* communities of practice [37] are good examples of existing platforms that could be used for these ends.

Second, there is a general consensus that most countries do not have sufficient technical expertise with IR methods and that this lack of experience and human resources is a significant barrier to accelerating action. The paucity is especially pronounced in low and middle-income countries. A growing collection of international tools are being developed that can support nascent technical teams – including the Food Environment Policy Index (FEPI) and the SEED tool mentioned above – however there remains an irreducible core set of skills that countries need to develop if they are to make use of these tools. Local investment in training, universities, and partnerships with other industries, sectors, and countries are vital to developing a domestic stock of technical expertise that can be leveraged for NCD policy implementation research.

A third issue is redressing the refractory underinvestment in IR from health research funders. Unfortunately, traditional NCD research funders have appeared reticent to finance IR capacity development. This may be because it is felt to stray too far from the funders’ core briefs, and it may also – understandably – stem from the perception of relatively high risks associated with convening multiple stakeholders to tackle complex issues that are deeply vested with political and commercial interests. In any case, governments and international public health organisations should be using whatever means they have at their disposal to incentivise greater investment in this sphere, as well as using their own pump-priming funds. In some settings it may be possible to use existing NCD funds to nurture capacity building activities, however there is still a lack of knowledge around *how best* to build and sustain national IR capacity. The US Centres for Disease Control and Prevention are approaching IR capacity building by focusing on improving “motivation, infrastructure, skill sets, training and funding, as well as bringing together both research and practice experts through initiatives such as leadership academies and communities of practice to share experiences and facilitate peer learning” [33]. Almost ironically, further implementation research will be required to understand how and why these initiatives to build IR capacity do or don’t work in different contexts.

Finally, capacity building requires more than hiring and training researchers in generic IR methods. The heart of IR research is interdisciplinarity and multisectorality. Strong IR work requires engagement of representatives from government, academia, the private sector and civil society to optimise NCD policy implementation. Countries therefore require personnel with skills and experience in working across and convening different disciplines. On a more fundamental level, a paradigm shift is required to establish strong and productive platforms and working relationships between researchers and policymakers. Whilst the research community has already established robust relationships with funders, policymakers are rarely directly involved in research activities. IR researchers addressing NCDs will need to engage with policymakers from multiple government departments including agriculture, sport, finance, transport, planning, education, business, employment, and housing.

Whilst working collaboratively across spheres and disciplines is indispensable, it is important to acknowledge that these relationships add layers of complexity, especially around leadership, goals and expectations, and the risks and rewards involved when partnering with industry. The NCD research community still lacks the platforms or experience to engage with this essential work. And yet, the opportunity to leverage multiple stakeholders for IR remains a major untapped opportunity. An example of a recent success comes from a Chinese collaboration between researchers, health policymakers and private companies to tackle the issue of high out-of-pocket costs for cancer medications. Roche and the large insurer *Swiss Re* have provided statistical analyses and reinsurance in a project that encouraged local firms to extend health insurance to 50 million Chinese citizens [38].

## Conclusions

We know what works in the fight against NCDs, but we don’t necessarily know *how* to implement the best-buy policies in many settings because complex contextual issues first need to be identified and overcome. There is a growing recognition among NGOs, governments, and academics that IR offers a powerful approach for translating policy solutions into effective action, however significant barriers still stand in the way. We identify four main areas for action: high-level engagement with IR among international NCD leaders; domestic investment in IR technical capacity-building; the creation of new financing streams for IR research; and the development of multi-stakeholder engagement mechanisms that can convene and leverage the perspectives and resources of multiple actors with overlapping aims. Efforts to trial different approaches in each of these domains should themselves be subjected to IR in order to understand the contextual factors that influence success. There are ripe opportunities for cross-country learning and collaboration, and WHO is well-placed to play a coordinating role. Whilst IR will not solve the NCD crisis, its spread and scale can help accelerate much-needed progress to tackle NCDs.

## Supplementary information

**Additional file 1.**

## Data Availability

Original PMAC report available upon request.
