# Supplementary material for: Unleashing implementation research to accelerate national noncommunicable disease responses
Source: Global Health. 2022 Jan 24;18:6. doi: 10.1186/s12992-021-00790-5 (PMC8785572; doi:10.1186/s12992-021-00790-5)
Supplement: Supplementary file 1 — Additional file 1. [file 12992_2021_790_MOESM1_ESM.docx]

# Appendix

**Priorities identified for the Malaysian national healthy diet strategy:**

- Enact a policy to restrict unhealthy food and beverage marketing in children’s settings and media;
- Mandate nutrition labelling (sodium and total sugars) and require caloric menu board labelling for all fast food chain outlets;
- Set sodium targets and investigate food composition standards for added sugars and saturated fat;
- Investigate restriction on opening hours of fast food restaurants and seek opportunities to restrict new placement near schools and residential areas;
- Introduce taxes on sugary drinks with revenues applied to healthy diets for children and investigate the price rise in fruits and vegetables;
- Continue to designate funding for research, with the budget commensurate with size of the health burden from unhealthy diets;
- Optimize the usage of existing monitoring system (SEGAK Data) and provide appropriate feedback and referral mechanism;
- Strengthen access to information related to public consultation and provide open access for submission by the main affected parties.
